# Supplementary material for: WordCommentsAnalyzer: A windows software tool for qualitative research
Source: F1000Res. 2018 Sep 4;7:536. Originally published 2018 May 3. [Version 2] doi: 10.12688/f1000research.14819.2 (PMC6137411; doi:10.12688/f1000research.14819.2)
Supplement: Supplementary file 3 [file f1000research-7-17614-s0002.tgz › b2a79496-4ada-47c4-9682-a99ce867836d.zip!/Eight Iranian Health Journals/Journal of Health System Research _38.docx]

© Journal of Health System Research . This work is licensed under CC BY-NC 4.0 (https://creativecommons.org/licenses/by-nc/4.0/).

Volume & Issue: دوره 8، شماره 1؛ 1391

Title: اثر نمايه گلايسميک رژيم غذايي بر عملکرد ورزشي ورزشکاران

http://hsr.mui.ac.ir/index.php/jhsr/article/view/539

يکي از عوامل تأثيرگذار بر موفقيت و افزايش عملکرد ورزشي ورزشکاران، کيفيت و کميت کربوهيدرات رژيم غذايي است. بنابراين، نقش GI (Glycemic index) در مطالعات متعدد، به منظور بررسي انواع مختلف کربوهيدرات‌هاي مصرفي مورد بررسي قرار گرفته است و اثرات آن در کاهش يا بهبود عملکرد ورزشي نشان داده شده است. هدف از انجام اين مطالعه مروري بر مطالعات جديد انجام شده در زمينه اثر مصرف کربوهيدرات‌هاي مصرفي با شاخص‌هاي گلايسميک متفاوت بر عملکرد ورزشي است. جستجوي انجام شده در بانک اطلاعاتي PubMed با استفاده از کلمات کليدي Exercise و GI و بين فاصله سال‌هاي 2010-1990 در نمونه‌هاي انساني انجام شد. نتايج مصرف کربوهيدرات‌هاي با شاخص گلايسميک پايين در قبل از ورزش بر بهبود عملکرد ورزشکاران ضد و نقيض بود. همچنين مصرف مقدار کربوهيدرات مصرفي در طول ورزش، به عنوان عامل اثرگذارتري نسبت به وعده غذايي قبل از ورزش بر عملکرد ورزشي عنوان شد. اثر شاخص گلايسميک بالا بر بازسازي ذخاير گليکوژن پس از ورزش به صورت يکنواخت در مطالعات اثبات شد. مصرف کربوهيدرات‌هاي با نمايه گلايسميک بالا در حين ورزش و پس از آن بر بهبود عملکرد ورزشکاران همسو است. بنابراين به نظر مي‌رسد که بيشتر مطالعات آينده در اين خصوص بايد اثر چنين رژيم‌هاي را در قبل از ورزش مورد آزمون قرار دهند.

Title: اثرات باقی‌مانده نانولوله‌های کربنی مورد استفاده در تصفیه فاضلاب بر روی سلامتی انسان

http://hsr.mui.ac.ir/index.php/jhsr/article/view/530

از آن جا که تولید فاضلاب سال به سال افزایش می‌یابد و نیز به دلیل تنوع آلاینده‌های موجود در آن، روش‌های مؤثر و پیشرفته‌ای برای تصفیه آن مورد نیاز است. یکی از موادی که در سال‌های اخیر به صورت رو به رشد برای تصفیه فاضلاب استفاده می‌شود، نانولوله‌های کربنی است. تماس با باقیمانده این مواد (مشابه باقی‌مانده مواد شیمیایی) که در اثر بلع آب، تنفس و تماس پوستی در طول فعالیت‌های معمول در خانه (نظیر حمام کردن و پخت و پز) صورت می‌گیرد، ممکن است اثرات نامطلوب زیادی بر سلامت انسان گذارد. علیرغم اثرات بالقوه نانولوله‌های کربنی بر سلامتی انسان و محیط‌زیست و همچنین کاربرد بسیار زیاد این مواد، اطلاعات در مورد سمیت آن‌ها در آب آشامیدنی بسیار کم است. هدف از این مطالعه، پوشش اثراتی است که باقیمانده این مواد در آب آشامیدنی بر روی سلامت انسان می‌گذارد.

Title: مروری بر انواع خودکشی از دیدگاه سلامت

http://hsr.mui.ac.ir/index.php/jhsr/article/view/724

خودکشی یکی از پیچیده‌ترین جنبه‌های زندگی انسان می‌باشد که تمامی ابعاد و زوایای آن به درستی شناخته نشده است. با توجه به اهمیت موضوع و از آن جایی که شناخت دقیق انواع خودکشی می‌تواند در پیش‌گیری از این پدیده کمک نماید، در مقاله حاضر سعی شده است تا طبقه‌بندی جدیدی از انواع خودکشی از دیدگاه سلامت مورد بحث قرار گیرد. بر این اساس، خودکشی به انواع تک‌ نفره، دو نفره، دسته جمعی، کمک شده، مأموریتی و تجمع خودکشی تقسیم گردیده و خصوصیات هر یک در متن مقاله شرح داده خواهد شد.

Title: بررسی آلودگی میکروبی دکمه‌‌‌های آسانسور در دانشکده‌های دانشگاه علوم پزشکی اصفهان

http://hsr.mui.ac.ir/index.php/jhsr/article/view/534

مقدمه: کنترل میکروارگانیسم‌ها در سطوح مختلف در محیط‌های صنعتی و مسکونی به لحاظ اشاعه بیماری‌های عفونی از نگرانی‌های اصلی می‌باشد. هدف این مطالعه، بررسی آلودگی باکتریایی دکمه‌های آسانسور در دانشکده‌های دانشگاه علوم پزشکی اصفهان در تابستان 1390 است.  روش‌ها: این تحقیق به صورت توصیفی مقطعی صورت گرفت. پس از نمونه‌برداری از سه محیط کشت TSB، EMB (Environmental Management Bureau) و بلادآگار استفاده شد و در مرحله بعد، کشت در مراحل تکمیلی ادامه داشت. یافته‌ها: حضور باکتری‌های انتروباکتر، سراتیا، انتروباکتر هافنیا و استافیلوکوک مشاهده شد. آسانسورهای دانشکده دندانپزشکی 100 درصد آلوده به این باکتری‌ها بودند که با عنایت به اقتضای فعالیت‌های افراد در این محیط‌ها، مواد و زائدات تولیدی قابل انتظار است. نمونه‌های گرفته شده از دانشکده مدیریت و پزشکی نیز 60 درصد آلوده به این باکتری‌ها بودند. آلودگی در بقیه دانشکده‌ها، 30 درصد بود. نتیجه‌گیری: این مطالعه نشان داد که در دانشکده‌‌های دارای آزمایشگاه به خصوص آزمایشگاه‌ها و پایلوت‌های بیولوژیکی، آلودگی و تنوع باکتریایی بیشتر است. در این میان باکتری‌های گرم منفی حضورشان محتمل‌تر است و باکتری‌های گرم مثبت در این سطوح فلور‌پوستی هستند. آلودگی و تنوع میکروبی در دکمه‌های آسانسور وجود داشت و در صورت عدم توجه به بهسازی محیط و رعایت بهداشت فردی، احتمال افزایش آلودگی در این اماکن وجود دارد. از این رو توجه جدی، آگاه‌سازی و آموزش عمومی ضروری است.

Title: بررسی ارتباط شاخص توده بدنی و حاملگی ناخواسته در زنان

http://hsr.mui.ac.ir/index.php/jhsr/article/view/536

مقدمه: رشد جمعیت ایران در دو دهه اخیر چنان افزایش یافته است که در مقاطعی به رشد انفجاری رسیده است. اکنون پیامدهای این رشد جمعیت به خوبی محسوس است و در آینده هم مدت‌ها ادامه خواهد یافت. روش‌های مختلفی برای تنظیم خانواده وجود دارد و یکی از مشکلاتی که در این زمینه وجود دارد، حاملگی ناخواسته با وجود استفاده از قرص‌های پیشگیری از بارداری است. با وجود تعداد بالای بارداری‌های مرتبط با مصرف قرص‌های ضدبارداری، بررسی‌ها برای ریسک فاکتورهای بیولوژیک دخیل در نارسایی این روش محدود است. یکی از ریسک فاکتورهای ممکن وزن بدن می‌باشد. با توجه به شیوع روز افزون چاقی در زنان و با توجه به این که مطالعات در این زمینه ناکافی است، بررسی‌هایی در این زمینه ضروری است. روش‌ها: این پژوهش یک مطالعه توصیفی– تحلیلی گذشته‌نگر است که بر روی 202 زن در سنین باروری با میانگین سنی 30 سال صورت گرفت. به طوری که با استفاده از نمونه‌گیری خوشه‌ای از بین 49 مرکز بهداشتی درمانی شهرستان لنجان، 20 مرکز انتخاب شد. در هر مرکز 20 پرونده به طور تصادفی انتخاب شد. سپس اطلاعات لازم شامل خواسته یا ناخواسته بودن حاملگی، سن، تحصیلات، تعداد زایمان‌ها، فاصله بین فرزندان، شاخص توده بدنی و مدت زمان استفاده از قرص‌های ضدبارداری از طریق مصاحبه با افراد و مشاهده پرونده افرادی که شرایط ورود به مطالعه را داشتند، تکمیل گردید. اطلاعات توسط نرم‌افزار SPSS و آزمون آماری رگرسیون لجستیک آنالیز گردید و نتایج مورد تجزیه و تحلیل قرار گرفت. یافته‌ها: در بین افرادی که از قرص استفاده کردند و باردار شدند، 1/82 درصد حاملگی خواسته و 9/17 درصد حاملگی ناخواسته داشتند. از نظر شاخص توده بدنی 1/1 درصد از این زنان BMI (Body mass index) کمتر از 5/18 داشتند که لاغر محسوب می‌شدند. 56/39 درصد از نظر وزنی طبیعی بودند. 8/47 درصد دارای اضافه وزن و 5/11 درصد چاق بودند. از نظر مدت زمان استفاده از قرص‌های ضدبارداری 4/39 درصد کمتر از 12 ماه و 5/18 درصد بالای 49 ماه استفاده کردند. آزمون t-test نشان داد که میانگین شاخص توده بدنی قبل از بارداری و زمان بارداری در افرادی که حاملگی ناخواسته داشتند به طور معنی‌داری بیشتر است (004/0 = P و 008/0 = P). نتیجه‌گیری: با توجه به نتیجه به دست آمده و عوارض حاملگی ناخواسته که شامل افزایش مرگ و میر مادر و جنین، ناهنجاری در جنین، سقط ناسالم و غیر بهداشتی می‌باشد، توجه به تغییر روش پیشگیری از بارداری یا تغییر دوز قرص‌های ضدبارداری در زنان چاق ضروری به نظر می‌رسد.

Title: شناسایی گونه‏های میکروبی هوازی تجزیه‌کننده بی‏فنیل‏های چند کلره

http://hsr.mui.ac.ir/index.php/jhsr/article/view/538

مقدمه: بی‏فنیل‏های چند کلره PCBs (Polychlorinated biphenyl)، گروهی از ترکیبات آروماتیک مصنوعی هستند که شامل دو حلقه بنزن و دارای یک تا ده اتم کلر هستند. وجود این ترکیبات در محیط‌زیست به دلیل سمیت، پایداری و همچنین خاصیت تجمع بیولوژیکی برای سلامتی مخاطره آمیز است و بایستی قبل از تخلیه به محیط‌زیست تصفیه شوند چرا که باعث آلودگی آب‏، خاک و هوا خواهند شد. تجزیه میکروبی PCBs به عنوان یکی از روش‏های مقرون به صرفه و کارامد برای حذف این ترکیبات از محیط مورد توجه است. هدف از این مطالعه شناسایی گونه‏های میکروبی هوازی تجزیه‌کننده PCBs می‏باشد. روش‏ها: این مطالعه با استفاده از راکتور SBBR (Sequencing batch biofilm reactor) در مقیاس آزمایشگاهی انجام شد. داخل راکتور از فوم (Polyurethane) به عنوان مدیا استفاده شد. فاضلاب سنتتیک مورد استفاده جهت تغذیه راکتور شامل سوبستره کمکی (استون به عنوان حلال برای ترکیب PCBs)، سوبستره اصلی شامل روغن ترانسفورماتور حاوی PCBs به عنوان منبع کربن و همچنین میکروالمنت‏ها بود. پس از دستیابی به جمعیت میکروبی کافی و گذراندن مراحل سازگاری میکروارگانیسم‏ها با ترکیبات PCBs، مراحل شناسایی گونه‏های میکروبی تجزیه‌کننده PCBs با تعیین توالی ژن 16SrRNA باکتری‌های جداسازی شده انجام شد. یافته‏ها: تعیین توالی ژنی باکتری‏های جداسازی شده نشان داد که گونه‏هایRhodococcus ، Pseudomonas، Pseudoxanthomonas، Agromyces و Brevibacillus باکتری‏های غالب تجزیه‌کننده ترکیبات PCBs در مطالعه حاضر هستند.  نتیجه‏گیری: ترکییبات PCBs با وجود دارا بودن خواص پایداری شیمیایی و سمیت می‌توانند توسط بعضی از میکروارگانیسم‏ها تحت شرایط هوازی یا بی‌هوازی تجزیه شوند و یا حداقل به کانجنرهای دارای کلر کمتر تبدیل شوند. بر اساس نتایج، پنج گونه باکتریایی که توانایی تجزیه روغن‌های ترانسفورماتور حاوی ترکیبات PCB را دارا هستند، شناسایی گردیدند.

Title: تعیین پروفایل لیپیدها و آپولیپو پروتئین‌های سرم در دانش‌آموزان دختر مدارس ابتدایی شهرستان خمینی‌شهر در مقایسه با مقادیر مرجع و وضعیت تغذیه‌ای آن‌ها

http://hsr.mui.ac.ir/index.php/jhsr/article/view/540

مقدمه: تغییر در میزان چربی‌ها، لیپو پروتئین‌ها و آپولیپوپروتیئن‌های سرم می‌تواند به عنوان یک عامل خطر قوی برای بیماری‌های قلبی- عروقی محسوب شود (2، 1). مطالعه حاضر به بررسی سطح لیپیدها و آپولیپوپروتئین‌های سرم در دختران مدارس ابتدایی شهرستان خمینی‌شهر پرداخت تا عواملی که دانش‌آموزان در حال حاضر و یا احتمالاً در آینده در معرض خطر ابتلا به افزایش چربی خون و ناراحتی‌های قلبی- عروقی قرار خواهد داد، شناخته شود. روش‌ها: صد نفر از دانش‌آموزان دختر مدارس ابتدایی شهرستان خمینی‌شهر به طور تصادفی انتخاب شدند. در حالت ناشتا از آن‌ها نمونه خون گرفته شد. فرم اطلاعات شخصی، فرم موافقت شرکت در تحقیق و پرسش‌نامه یادآمد 24 ساعته برای سه روز (دو روز کاری و یک روز تعطیل) به والدین دانش‌آموزان داده شد که تکمیل کنند. سپس پرسش‌نامه‌ها تجزیه و تحلیل آماری شدند. مقادیر کلسترول تام، HDLc،TG (Triglyceride) و LDLc به روش آنزیمی و مقادیر 1APOA و 100APOB به روش ایمینوتوربیدومتری اندازه‌گیری شد. نتایج با مقادیر مرجع و وضعیت تغذیه‌ای مقایسه گردید. یافته‌ها: میانگین کلسترول تام (402/0 = P) و میانگین 1APOA (162/0 = P) با میانگین مرجع تفاوت معنی‌دار نداشت. میانگین TG (0001/0 = P)، HDLc (001/0 = P) و 100APOB (001/0 = P) به صورت معنی‌داری بالاتر از مقدار مرجع بود. میانگین LDLc (023/0 = P) به صورت معنی‌داری پایین‌تر از مقدار مرجع بود. بین پروتئین دریافتی از طریق غذا و مقدار 100APOB رابطه معنی‌دار و معکوس وجود داشت (212/0- = r، 034/0 = P). بین کلسترول دریافتی از طریق غذا و مقدار 1APOA رابطه معنی‌دار و مستقیم وجود داشت (250/0 = r، 012/0 = P). بین کربوهیدرات، فیبر و چربی‌های دریافتی از طریق غذا با متغیرهای مورد مطالعه رابطه معنی‌دار وجود نداشت. نتیجه‌گیری: مقدار کلسترول تام در این دانش‌آموزان نرمال بود که با نتایج تحقیقات دیگر که در آمریکا و ژاپن روی بچه‌ها انجام شده بود، هماهنگی داشت. پس مصرف چربی‌های جانوری را برای آن‌ها محدود نمی‌شود و در حد نیاز باید مصرف کنند. مقدار TG در این دانش‌آموزان بالاتر از مقدار مرجع بود چرا که 50 درصد انرژی آن‌ها از کربوهیدرات تأمین شده بود. پس مقدار TG آن‌ها نرمال باشد اما به علت مصرف زیاد کربوهیدرات تری‌گلیسیرید آن‌ها بالا بود . بنابراین توصیه می‌شود غذاهای دریایی و کربوهیدرات‌های پیچیده، سبزیجات و میوه‌جات را بیشتر مصرف کنند. کاهش مقدار LDLC در این بچه‌ها به علت افزایش فعالیت فیزیکی و افزایش مصرف پروتئین‌های گیاهی نسبت به پروتئین‌های جانوری است. TG و HDLC رابطه معکوس با هم دارند. در این تحقیق با بالا بودن مقدار TG انتظار می‌رفت که مقدار HDLC پایین باشد که نتایج عکس این مطلب را تأیید کرد. بالا بودن مقدار HDLC به دلیل فعالیت فیزیکی زیاد در بچه‌هاست چرا که در یک منطقه نیمه روستایی هستند. بالا بودن مقدار HDLC و نرمال بودن مقدار 1APOA به دلیل افزایش مقدار 2HDL در مقایسه با 3HDL است که در پیشگیری از بیماری‌های قلبی- عروقی بازدارنده‌تر هستند. با توجه به کاهش LDLC و افزایش مقدار 100APOB به نظر می‌رسد که ذرات LDLC از نوع ذرات تیپ B کلسترول باشد؛ یعنی LDLC با سایز کوچک که آتروژن است و نفوذپذیری بیشتری به درون عروق خونی دارد. از طرف دیگر افزایش TG باعث افزایش 100APOB می‌باشد. هیچ ارتباط معنی‌داری بین کربوهیدرات، فیبر و چربی‌های دریافتی از طریق غذا با متغیرهای مورد مطالعه وجود نداشت.

Title: بررسی ارتباط سلامت عمومی با اعتیاد به اینترنت در دانشجویان دانشگاه علوم پزشکی اصفهان

http://hsr.mui.ac.ir/index.php/jhsr/article/view/541

مقدمه: اینترنت یکی از منابع مهم جهت دسترسی به متون علمی در دانشگاه‌های علوم پزشکی محسوب می‌شود و دانشجویان، از آن جهت برآوردن اهداف حرفه‌ای و شخصی خود استفاده می‌کنند. با توجه به این که میزان بروز و شیوع اعتیاد به اینترنت در این گروه در حال افزایش است، این مطالعه با هدف، تعیین ارتباط سلامت عمومی با اعتیاد به اینترنت در دانشجویان دانشگاه علوم پزشکی اصفهان در سال 1389 انجام گرفت. روش‌ها: در این مطالعه مقطعی، 123 نفر از دانشجویان دانشگاه علوم پزشکی اصفهان به صورت تصادفی انتخاب شدند. از دو پرسش‌نامه سنجش سلامت عمومی و اعتیاد به اینترنت Yang جهت جمع‌آوری داده‌ها استفاده گردید. در نهایت امتیازهای افراد به دو دسته مساوی و پایین‌تر از 49 و بالاتر از 50 تقسیم شد که به ترتیب نشان دهنده وضعیت کاربران به صورت عادی و کاربران معتاد می‌باشد. اطلاعات گرد آمده توسط نرم‌افزار 16SPSS و آزمون‌های Independent t-test و kruskal-Walis مورد تجزیه و تحلیل قرار گرفت. یافته‌ها: 5/80 درصد دانشجویان نرمال و 5/19 درصد اعتیاد خفیف داشتند. میانگین سلامت جسمی، اختلال در عملکرد اجتماعی، اضطراب و افسردگی در گروه معتاد به اینترنت به ترتیب 77/3 ± 28/15، 16/4 ± 69/15، 40/4 ± 67/10 و 21/6 ± 42/7 به دست آمد. بین ابعاد سلامت عمومی در هر دو گروه رابطه آماری معنی‌داری مشاهده شد (01/0 > P). نتیجه‌گیری: با توجه به تأثیر اعتیاد به اینترنت بر ابعاد سلامتی دانشجویان، نظارت و کنترل بر استفاده از اینترنت در دانشجویان دانشگاه و همچنین آگاه‌سازی آنان از اثرات مخرب استفاده ناصحیح از اینترنت ضروری به نظر می‌رسد.

Title: تجربیات و دیدگاه‌های زنان و متخصصان زنان و زایمان در خصوص انتخاب روش زایمان: یک مطالعه کیفی

http://hsr.mui.ac.ir/index.php/jhsr/article/view/542

مقدمه: طبق توصیه سازمان بهداشت جهانی محدوده قابل قبول برای سزارین تنها 15-10 درصد از کل زایمان‌های انجام شده می‌باشد (1). افزایش میزان سزارین از این محدوده می‌تواند سبب افزایش مرگ و میر و عوارض در مادران و نوزادان گردد. آمارهای به دست آمده از استان‌ها و شهرهای ایران بیان‌گر رشد روزافزون سزارین‌های غیر ضروری در ایران می‌باشند. روش‌ها: در این مطالعه کیفی به بررسی نظرات تعدادی از مادرانی که تحت عمل جراحی سزارین انتخابی و اورژانسی قرارگرفته بودند (در بیمارستان مهر اهواز، بیمارستان امام خمینی و بیمارستان رازی) و همچنین جمعی از متخصصین زنان و زایمان شهر اهواز و ماه‌شهر، پرداخته شد. مصاحبه‌های نیمه ساختار یافته تا رسیدن به اشباع داده‌ها ادامه یافت. اطلاعات به دست آمده با استفاده از آنالیز محتوا مورد تجزیه و تحلیل قرار گرفت. یافته‌ها: مادرانی که به درخواست خودشان تحت عمل سزارین قرار گرفته بودند، به دلیل ترس از زایمان طبیعی به سمت سزارین گرایش پیدا کرده‌اند و سزارین را روش بی‌درد و بی‌خطری برای خود و نوزادشان می‌دانستند. در مقابل مادرانی که به صورت اورژانسی عمل سزارین انجام داده بودند، این روش را از دوران بارداری به عنوان روش ارجح پذیرفته بودند و درد آن ‌را مقطعی دانسته بودند و بهبودی سریع‌تر پس از زایمان طبیعی را مزیت مهم آن می‌دانستند. پزشکان نیز تمایل بیمار و همکاری وی را به عنوان فاکتور اثرگذار ذکر نمودند. در کنار آن به مسايل قانونی و امنیتی، بعد مالی و اهمیت زمان اشاره نمودند. نتیجه‌گیری: مطابق نتایج این بررسی، برای ترغیب مادران به انجام زایمان طبیعی نیاز است که در دوران بارداری آمادگی‌های لازم فیزیکی و روانی در آنان ایجاد شود.  همچنین به خطرات سزارین و در مقابل مزیت‌های زایمان آگاهی یابند، تا در نهایت انتخاب بهتری بنمایند. از طرف دیگر مسؤولین به رفع نقایص و ایجاد شرایط مناسب جهت انجام زایمان طبیعی در زایشگاه‌ها توجه کافی نمایند.

Title: عوامل مؤثر بر رضایت شغلی کارکنان بیمارستان‌های آموزشی- درمانی شهر همدان

http://hsr.mui.ac.ir/index.php/jhsr/article/view/543

مقدمه: موفقیت هر سازمان بستگی به تلاش و رضایت شغلی کارکنان آن دارد. هدف از انجام این مطالعه تعیین عوامل مؤثر بر رضایت‌مندی شغلی پرسنل بیمارستان‌های آموزشی- درمانی شهر همدان بود. روش‌ها: این پژوهش یک مطالعه توصیفی و از نوع مقطعي (Cross-sectional) بود که بر روي 350 نفر از کارکنان پنج بیمارستان آموزشی- درمانی شهر همدان با نمونه‌گیری طبقه‌بندی تصادفی انجام گرفت. اطلاعات به وسیله پرسش‌نامه استاندارد به صورت خود گزارش‌دهی جمع‌آوری شد. داده‌ها در نرم‌افزار آماری 13SPSS و با بهره‌گیری از آزمون‌های آماری 2χ، Independent t-test، ضریب همبستگی Pearson و Linear regression analyzes مورد تجزیه و تحلیل قرار گرفت. یافته‌ها: از نظر رضایت شغلی 5/6 درصد به طور کامل ناراضی، 9/59 درصد ناراضی، 9/30 درصد راضی و 7/2 درصد به طور کامل راضی بودند. Linear regression analyzes نشان داد که فاكتورهاي سرپرستی، ماهیت کار و حقوق به میزان بیشتری در میزان رضایت شغلی به طور کلی نقش داشته‌اند. همچنین بین افزایش سن، سابقه کاری و کاهش رضایت شغلی رابطه آماری معنی‌داری دیده شد (05/0 = P). نتیجه‌گیری: نتایج نشان داد که توجه به موضوع سرپرستی در سازمان همراه با ایجاد جوی مناسب در محیط کار و همچنین تلاش برای برطرف کردن مشکلات اقتصادی کارکنان در افزایش رضایت شغلی آنان مؤثر می‌باشد.

Title: ارزیابی پتانسیل خورندگی و یا رسوب‌گذاری در منابع تأمین، مخزن و شبکه توزیع آب شهر کیان (استان چهارمحال و بختیاری) با استفاده از دو شاخص لانژلیه و رایزنر

http://hsr.mui.ac.ir/index.php/jhsr/article/view/544

مقدمه: خوردگی را می‌توان به عنوان انهدام یک فلز طی یک واکنش شیمیایی تعریف کرد. به بیان دیگر خوردگی، اندازه‌گیری تهاجم آب در لوله‌ها و تجهیزات زنگ زده می‌باشد. در گذشته همه تحقیقات بر روی نشت و خسارت اقتصادی حاصل از خوردگی متمرکز شده است. در حال حاضر علاوه بر جنبه‌های اقتصادی، جنبه‌های بهداشتی و زیباشناختی ناشی از پدیده خوردگی نیز مدنظر قرار گرفته است. این تحقیق با هدف تعيين ميزان استعداد خورندگي منابع آب شرب شهری با استفاده از روش‌های محاسباتی انجام گرفت. سیستم آب‌رسانی شهر کیان در استان چهارمحال و بختیاری به عنوان الگو انتخاب شد. روش‌ها: با در نظر گرفتن تراکم جمعیت، مساحت منطقه و نقشه آب‌رسانی شهرک، تعداد ٧ نقطه جهت انجام نمونه‌برداری تعیین گردید. عملیات نمونه‌برداری و پس از آن آنالیزهای آزمایشگاهی انجام شد. پارامترهای درجه حرارت، سختی کلسیم، قلیاییت، کل جامدات محلول و pH اندازه‌گیری شد و اندیس‌های خورندگی محاسبه گردید. علاوه بر این، پارامترهای CO2، یون سولفات و کلراید نیز که در میزان خورندگی مؤثر می‌باشند، اندازه‌گیری شد. کلیه آزمایش‌ها بر اساس کتاب روش‌های استاندارد جهت آزمایش‌ها آب و فاضلاب چاپ بیستم صورت گرفت.   یافته‌ها: کلیه پارامترهای مؤثر در براورد شاخص‌های خورندگی به استثنای میزان متوسط دما و pH، خارج از حدود استاندارد بودند. محاسبات مربوط به اندیس‌های خورندگی لانژلیه و رایزنر نشان داد که میزان متوسط خورندگی به ترتیب برابر ٦۸/٠- و ٥٢/٨ بود. این ویژگی به تدریج در طول شبکه افزایش یافت. همچنین بیشترین میزان اندیس‌های خورندگی مربوط به فصول پاییز و زمستان بود. نتایج حاصل از انجام آنالیزهای آماری نشان داد که ٧٢ درصد از نمونه‌هایی که از نظر شاخص لانژلیه در رده خوردگی جزیی قرارگرفتند، از نظر شاخص رایزنر در رده خوردگی شدید قرار می‌گیرند. بحث و نتیجه‌گیری: نتایج نشان داد که آب شهرک کیان دارای خورندگی (با شرایط جزیی تا شدید) می‌باشد. همچنین نتایج این تحقیق نشان داد که در راستای تصمیم‌گیری جامع‌تر و دقیق‌تر، بهتر است از دو شاخص خورندگی لانژلیه و رایزنر به همراه یکدیگر استفاده شود. لازم به ذکر می‌باشدکه کاربرد این روش‌ها در مقایسه با روش‌های دیگر از سادگی بیشتری برخوردار می‌باشد.

Title: بررسی کاربرد فرایندهای انعقاد و اسید کراکینگ در بهبود قابلیت تجزیه بیولوژیکی فاضلاب صنعت روغن‌کشی زیتون

http://hsr.mui.ac.ir/index.php/jhsr/article/view/545

مقدمه: فاضلاب صنعت روغن زیتون بسته به محتویات شیمیایی و مواد آلی مقاوم به تجزیه بیولوژیکی است. به علت وجود ترکیبات بازدارنده فنولی، سمیت برای میکروارگانیسم‌ها در واحدهای تصفیه فاضلاب شهری، بوی نامطبوع، COD (Chemical oxygen deman) و BOD (Biochemical oxygen demand) بالا، جامدات معلق بالا، ترکیبات مقاوم به تجزیه بیولوژیکی، تهدیدی برای منابع آب سطحی و زیرزمینی محسوب می‌گردد. هدف از این مطالعه نیز، تعیین کارایی فرایندهای تلفیقی انعقاد و اسید کراکینگ جهت کاهش بار آلودگی و بهبود قابلیت تجزیه‌پذیری بیولوژیکی فاضلاب روغن زیتون بود. روش‌ها: این مطالعه در مقیاس آزمایشگاهی و به صورت ناپیوسته بر روی فاضلاب واقعی انجام شد. فرایند انعقاد با استفاده از مواد منعقد کننده آلوم و کلرور‌فریک و بر روی پساب حاصل از عملکرد بهترین ماده منعقد کننده انجام گرفت. فرایند اسید کراکینگ با استفاده از اسید سولفوریک انجام پذیرفت. میزان حذف پارامترهای COD، کل ترکیبات فنلیک و TSS (Total suspended solids) مورد بررسی قرار گرفت. يافته‌ها: بالاترین راندمان حذف آلاینده‌ها در PH و دوز بهینه کلرورفریک به ترتیب 6 و mg/l 3000 حاصل گردید. در این شرایط میزان حذف COD، ترکیبات فنلی و TSS توسط کلرورفریک به ترتیب 2/91، 7/89 و 9/98 درصد حاصل گردید. همچنین ماده منعقد کننده کلرور‌فریک نسبت به آلوم برتری داشت. فرایند اسیدکراکینگ بر روی پساب حاصل از عمل انعقاد در شرایط بهینه به ترتیب باعث حذف 6/18 و 6/52 درصد COD و کل ترکیبات فنلی گردید. همچنین اندازه‌گیری نسبت BOD/COD نشان داد که این مقدار بعد از فرایند تلفیقی از 14/0 به 69/0 افزایش یافته است. نتيجه‌گيري: این مطالعه نشان داد که این فرایندها می‌توانند به عنوان روش‌های پیش‌تصفیه‌ مؤثر و اقتصادی در کاهش بار آلایندگی این نوع فاضلاب به کار گرفته شود.

Title: عوامل مؤثر بر آگاهي، نگرش و عملكرد محققین دانشگاه علوم پزشکی گلستان در مورد انتقال دانش حاصل از پژوهش

http://hsr.mui.ac.ir/index.php/jhsr/article/view/549

مقدمه: در سال‌هاي اخير انتقال دانش و استفاده از شواهد پژوهش براي ارتقاي سلامت در سطح جهاني بسيار مورد توجه قرار گرفته است. هدف از اين مطالعه، بررسي عوامل مؤثر بر ميزان آگاهي، نگرش و عملكرد محققين دانشگاه در مورد انتقال دانش حاصل از پژوهش در سال 1389 بود. روش‌ها: در اين مطالعه مقطعي، از مجموع 120 پژوهش‌گر واجد‌الشرايط دانشگاه علوم پزشكي گلستان، 89 محقق پرسش‌نامه را پاسخ دادند. ابزارهاي جمع‌آوري داده‌ها شامل پرسش‌نامه آگاهي، نگرش و عملكرد محققين در زمينه انتقال دانش حاصل از پژوهش بود. حيطه‌هاي مورد نظر در پرسش‌نامه شامل بخش متغيرهاي فردي، درصد زمان اختصاص داده به فعاليت‌هاي پژوهشي از كل فعاليت‌ها، بخش آگاهي (4 سؤال، دامنه نمرات 4-0)، نگرش (14 سؤال، دامنه نمرات 56-0)، عملكرد (8 سؤال) و امتياز خود‌ارزيابي انتقال دانش (1 سؤال، دامنه نمرات 10-0) بود. داده‌ها با استفاده از آزمون‌هاي تحليلي Linear regression و ضريب همبستگي Spearman مورد تجزيه و تحليل قرار گرفت. يافته‌ها: ميانگين نمره آگاهي شركت كنندگان در زمینه انتقال دانش 92/1 (با انحراف معيار 97/0)، ميانگين نمره نگرش 70/28 (با انحراف معيار 40/5) و در زمينه عملكرد ميانگين تعداد مقالات (داخلي و خارجي) پژوهش‌گران در دو سال اخير 50/4 (با انحراف معیار 97/4)، ميانگين تعداد طرح‌های پژوهش‌گران (مجری يا همکار) 02/5 (با انحراف معیار 41/4) به دست آمد. امتياز آگاهي با سن (28/0- = ضريب همبستگي، 008/0 = P) و سال‌هاي اشتغال (27/0- = ضريب همبستگي، 01/0 = P) رابطه معكوس معني‌دار داشت؛ در حالي كه ميانگين نمره نگرش با هيچ يک از متغيرهاي مستقل رابطه معني‌داری نداشت (05/0 < P). میانگین امتیاز خود‌ارزيابي محققين از فعاليت انتقال دانش با درصد ميزان زمان اختصاص يافته به فعاليت پژوهشی ارتباط مستقيم معني‌دار (028/0 = ضريب بتا، 01/0 = P) داشت. نتيجه‌گيری: نتايج مطالعه فعلي گوياي پايين بودن امتياز آگاهی، نگرش و عملكرد پژوهش‌گران در زمینه مفاهيم انتقال دانش حاصل از پژوهش بود.

Title: بررسی ارتباط بین سلامت ‌معنوی با استرس، اضطراب و برخی متغیرهای جمعیت‌شناختی زنان مبتلا به سرطان‌ سینه مراجعه کننده به مرکز درمانی تخصصی سرطان اصفهان

http://hsr.mui.ac.ir/index.php/jhsr/article/view/334

مقدمه: استرس و اضطراب از جمله اختلالات شایع و تأثیرگذار بر فرایند درمان در مبتلایان به سرطان‌ سیـنه می‌باشد. وجود ارتباطات پارادوکس‌گونه بین سلامت‌ معنوی با استرس و اضطراب در مبتلایان به سرطان از جمله سرطان ‌سینه در مطالعات خارجی و کمبود مطالعات مشابه در داخل کشور، محققین را بر آن داشت تا مطالعه‌ای را با هدف، ارتباط‌ بین سلامت‌ معنوی‌ با استرس، اضطراب و برخی متغیرهای جمعیت‌شناختی زنان مبتلا به سرطان سینه مراجعه ‌کننده به مرکز درمانی‌ تخصصی ‌سرطان ‌اصفهان در سال 1390 طراحی و اجرا نمایند‌. روش‌ها: مطالعه حاضر از نوع توصیفی- همبستگی بود که به شکل مقطعی اجرا گردید. نمونه‌های مورد پژوهش در این مطالعه متشکل از 266 نفر از زنان مبتلا به سرطان سینه بودند که با روش نمونه‌گیری آسان و از نوع در دسترس وارد مطالعه شدند. ابزار گردآوری اطلاعات در این مطالعه شامل فرم اطلاعات دموگرافیک، پرسش‌نامه استاندارد سنجش استرس، اضطراب و افسردگی 42-DASS (The depression, anxiety and stress scale) و پرسش‌نامه سلامت معنوی Ellison-Paloutzian بود. داده‌ها پس از جمع‌آوری با استفاده از نرم‌افزار آماری 18SPSS و روش‌های آمار توصیفی (توزيع درصد فراواني، ميانگين، انحراف معيار) و تحلیلی (آزمون t-student و ضریب همبستگی Pearson و Spearman)، مورد تجزیه و تحلیل قرار گرفت. یافته‌ها: سطح سلامت معنوی اکثریت واحدهای پژوهش (64/58 درصد) در محدوده متوسط و پس از آن در محدوده بالا (35/41 درصد) قرار داشت. میانگین نمره بعد مذهبی سلامت ‌معنوی بیماران (22/7 ± 52) بالاتر از میانگین نمره بعد وجودی سلامت ‌معنوی (67/9 ± 19/43) بود. ضریب همبستگی Pearson نشان داد بین استرس بیماران با تمامی ابعاد سلامت‌ معنوی ارتباط آماری معنی‌دار وجود دارد. همچنین یافته‌ها نشان داد بین اضطراب با نمره کلی سلامت‌ معنوی و بعد وجودی آن ارتباطی معنی‌دار وجود دارد، لیکن بین بعد مذهبی سلامت ‌معنوی و اضطراب ارتباطی وجود نداشت. یافته‌ها حاکی از عدم وجود هر گونه ارتباط معنی‌دار بین سلامت ‌معنوی با متغیرهای جمعیت‌شناختی بود. نتیجه‌گیری: این مطالعه نشان ‌داد که در مبتلایان به سرطان‌ سینه با نمرات‌ بالاتر سلامت‌ معنوی، میزان اضطراب و استرس کمتری وجود دارد، لذا پرداختن به تمامی ابعاد این بیماران از جمله بعد معنوی در روند درمان توصیه می‌شود. همچنین با توجه به عدم وجود ارتباط بین سلامت معنوی با متغیرهای جمعیت‌شناختی در مطالعه حاضر و وجود یافته‌های متناقض در این زمینه در سایر مطالعات، تحقیقات بیشتر الزامی است.

Title: تحلیل ارتباط بین میزان فلزات سنگین در نمونه‌های آب و رسوب تالاب انزلی

http://hsr.mui.ac.ir/index.php/jhsr/article/view/262

مقدمه: رسوبات ظرفيت بالايي در پيوند با فلزات سنگين آب‌هاي آلوده دارند. به دليل تغيير شرايط محيط‌هاي آبي، اين گونه رسوبات همواره به عنوان يك منبع بالقوه آلودگي منابع آب محسوب مي‌شوند. تالاب انزلی در حاشيه جنوبي درياي خزر به عنوان يك محيط آبي كه از جنبه اكوسيستم داراي اهميت بالايي مي‌باشد، همواره در معرض ورود آلودگي فلزات سنگين قرار داشته است. هدف از اين تحقيق، بررسي ميزان اثرپذيري رسوبات كف تالاب از آلودگي‌ فلزات سنگين محيط آبي فوقاني بود. روش‌ها: در اين تحقيق نمونه‌هاي آب و رسوب از كف تالاب انزلي در 12 ايستگاه برداشته شد و ميزان فلزات سنگين Pb، Ni، Cr، V و Hg در آن‌ها مورد اندازه‌گيري قرار گرفت. روش هضم كامل اسيدي جهت سنجش ميزان كل فلزات سنگين رسوبات مورد استفاده قرار گرفت. در ادامه جهت بررسي ارتباط بين ميزان عناصر در آب و رسوب از روش تحليل همبستگي كانونيك (Canonical correlation analysis یا CCA) استفاده گرديد. یافته‌ها: نتايج سنجش ميزان فلزات سنگين حاكي از غلظت بالاي فلزات سنگين به خصوص در نمونه‌هاي رسوب بود. اين مقادير براي سرب، نيكل، كروم، واناديوم و جيوه به ترتيب 5/19، 1/60، 1/90، 3/99 و 8/2 ميلي‌گرم در كيلوگرم وزن خشك خاك مشاهده گرديد. همچنين يافته‌هاي حاصل از CCA بيانگر وجود ارتباط قوي بين دو دسته ميزان عناصر در آب و رسوب بود. بررسي‌هاي بيشتر بر روي نتايج حاصل از CCA بيانگر تأثيرپذيري دسته پارامتر رسوب از عناصر كروم و جيوه و تأثيرپذيري دسته پارامتر آب از عناصر كروم، جيوه و سرب بود. نتیجه‌گیری: روش آماري مورد استفاده در اين تحقيق مي‌تواند به عنوان روشي مناسب و كم ‌هزينه در تحليل آلودگي محيط‌هاي آبي مورد استفاده قرار گيرد.

Title: آگاهی و نگرش زنان باردار نخست‌زای مراجعه کننده به مراکز بهداشتی درمانی شهری سمیرم در خصوص روش‌های زایمان: يك مطالعه مقطعي

http://hsr.mui.ac.ir/index.php/jhsr/article/view/722

مقدمه: زایمان طبیعی به عنوان روش ارجح ختم بارداری محسوب می‌شود، اما متأسفانه میزان زایمان طبیعی به دلیل افزایش زایمان سزارین رو به کاهش است. از جمله عواملی که منجر به انتخاب سزارین می‌شود، سطح آگاهی پایین از روش‌های ختم بارداری و نگرش منفی به زایمان طبیعی است. هدف از این مطالعه، بررسی آگاهی و نگرش زنان باردار نسبت به روش‌های زایمان می‌باشد كه در سال 1390 انجام گرفت. روش‌ها: مطالعه توصیفی- تحلیلی حاضر بر روی 104 زن باردار نخست‌زای واقع در سه ماهه سوم بارداری که به طور متوالی به مراکز بهداشتی درمانی شهری سمیرم مراجعه می‌کردند، به روش سرشماری انجام گردید. داده‌ها از طریق پرسش‌نامه‌ای شامل سؤالات دموگرافیک، آگاهی‌سنجی و نگرش‌سنجی جمع‌آوری شد و از طریق نرم‌افزار 16SPSS مورد تجزیه و تحلیل آماری قرار گرفت. در جمع‌آوری داده‌ها، روایی و پایایی پرسش‌نامه سنجیده شد. یافته‌ها: نتایج نشان داد، اکثریت افراد مورد پژوهش آگاهی در حد ضعیف (4/40 درصد) و متوسط (4/40 درصد) داشتند و 2/19 درصد از زنان باردار سطح آگاهی بالا داشتند. همچنین 7/32 درصد از زنان باردار نگرش مثبت، 7/57 درصد بی‌نظر و 6/9 درصد نگرش منفی به زایمان طبیعی داشتند. ارتباط معنی‌داری بین سطح آگاهی با سطح تحصیلات، نگرش و وضعیت شغلی وجود داشت، اما بین سطح آگاهی با سن، سن ازدواج و وضعیت اقتصادی ارتباط معنی‌داری وجود نداشت. ارتباط نوع نگرش با سن، سن ازدواج، سطح تحصیلات، وضعیت شغلی و وضعیت اقتصادی معنی‌دار نبود. نتیجه‌گیری: از آن جا که آگاهی اکثر زنان باردار نسبت به نوع زایمان در حد ضعیف و متوسط بود و همچنین اکثریت مادران در خصوص نگرش نسبت به زايمان طبيعي اظهار بی‌نظري كردند، بنابراين تأکید می‌گردد در مراکز بهداشتی درمانی به صورت معمول کلاس‌هاي آموزشی دوران بارداري، به ویژه برای زنان نخست‌زا تشکیل گردد و نظارت و پیگیري بر تشکیل کلاس‌ها و ارزیابی آن‌ها توصیه می‌شود.

Title: ارتباط بین ظرفیت حمل بار به روش NIOSH و خطر بروز اختلالات اسکلتی- عضلانی به روش Rapid upper limb assessment (RULA) در کارکنان سازمان بهزیستی شهر اصفهان

http://hsr.mui.ac.ir/index.php/jhsr/article/view/723

مقدمه: کارشناسان و کارکنان مراکز توان‌بخشی اغلب فعالیت‌های خود را بدون رعایت اصول ارگونومیکی و روش‌های صحیح حمل دستی بار انجام می‌دهند و از این رو به مرور زمان و با تکرار این فعالیت‌ها در معرض ابتلا به اختلالات اسکلتی- عضلانی قرار می‌گیرند. این پژوهش با هدف، بررسی ظرفیت حمل بار با استفاده از روش مؤسسه بین‌المللی ایمنی و سلامت شغلی (National institute for occupational safety and health یا NIOSH) و ارتباط آن با اختلالات اسکلتی- عضلانی در کارکنان سازمان بهزیستی شهرستان اصفهان انجام گرفت. روش‌ها: این مطالعه به صورت توصیفی- تحلیلی از نوع مقطعی بر روی 41 نفر از کارکنان مشاغل مختلف مراکز توان‌بخشی سازمان بهزیستی شهرستان اصفهان به روش سرشماری انجام شد. سپس ارزیابی وضعیت بدنی به روش ارزیابی سریع اندام فوقانی (Rapid upper limb assessment یا RULA) بر روی افراد این مشاغل صورت گرفت و در نهایت ظرفیت حمل بار این افراد به کمک معادله NIOSH محاسبه و داده‌ها با استفاده از آزمون‌های آماری همبستگی Spearman و Pearson و توسط نرم‌افزار 18SPSS مورد تجزیه و تحلیل قرار گرفت. یافته‌ها: آزمون همبستگی Spearman نشان داد که بین ظرفیت حمل بار و شدت اختلالات اسکلتی- عضلانی رابطه معنی‌داری وجود ندارد، اما بین میزان خمیدگی بدن و اختلالات اسکلتی- عضلانی ارتباط معنی‌داری به صورت مستقیم یافت شد (05/0 = P). همچنین آزمون همبستگی Pearson نشان داد که بین فاصله بار از بدن (09/0 = P) و قد فرد (37/0 = P) با ظرفیت حمل بار رابطه‌ای وجود ندارد. نتیجه‌گیری: شاید بتوان گفت ظرفیت حمل بار به طور مستقیم منجر به ایجاد اختلالات اسکلتی- عضلانی نمی‌شود، اما می‌تواند به طور غیر مستقیم و از طریق متغیرهای مؤثر بر ظرفیت حمل بار نظیر میزان خمیدگی و چرخش بدن در هنگام بلند کردن بار به ایجاد این اختلالات کمک کند.

Title: بررسی کارایی تصفیه‌خانه فاضلاب شرق اصفهان در دستیابی به شاخص انگلبرگ

http://hsr.mui.ac.ir/index.php/jhsr/article/view/725

مقدمه: امروزه کاربرد بازیافت فاضلاب در آبیاری محصولات کشاورزی مورد توجه زیادی قرار گرفته و این امر اهمیت آن را دو چندان کرده است که بالقوه می‌تواند مخاطرات بهداشتی و زیست محیطی جدی به همراه داشته باشد. لذا دستیابی به شاخص انگلبرگ جهت فاضلاب تصفیه شده ضرورت دارد. بر این اساس این تحقیق بر روی برکه‌های تثبیت تلفیقی تصفیه‌خانه شرق اصفهان به انجام رسید. روش‌ها: مطالعه توصیفی- مقطعی حاضر در فصل زمستان 1389 و بهار 1390 انجام گردید. از فاضلاب خام ورودی و پساب خروجی برای تعیین تعداد کلیفرم مدفوعی و از پساب خروجی برای تعیین تعداد تخم نماتود و به صورت لحظه‌ای و هر دو هفته یک بار نمونه‌برداری انجام و آزمایش‌ها مطابق کتاب روش استاندارد 2005 صورت گرفت. نتایج به دست آمده با استفاده از نرم‌افزار SPSS  و آزمون‌های آماری تجزیه و تحلیل شد. یافته‌ها: نتایج نشان داد که هیچ نوع تخم نماتودی در پساب خروجی در فصل‌های زمستان و بهار مشاهده نشد؛ در حالی که میزان کلیفرم مدفوعی و حذف آن در پساب خروجی به ترتیب در فصل زمستان،104 × 84/3 و 15/5 واحد لگاریتم و در فصل بهار،104 × 09/2 در 100 میلی‌لیتر و 5/4 واحد لگاریتم بود. میانگین هندسی تعداد کلیفرم‌های مدفوعی در فاضلاب خروجی تصفیه‌خانه در طول دوره104 × 9/2 در 100 میلی‌لیتر بود. در حذف کلیفرم‌های مدفوعی، اختلاف معنی‌داری بین دو فصل زمستان و بهار مشاهده نگردید (25/0 = P).   نتیجه‌گیری: با توجه به میانگین هندسی تعداد کلیفرم‌های مدفوعی در پساب خروجی در طول دوره و مقایسه با استاندارد اعلام شده از طرف سازمان بهداشت جهانی (1000 >)، استفاده از چنین پسابی جهت آبیاری مزارع و کشاورزی جهت آبیاری نامحدود مجاز نمی‌باشد.

Title: ارزیابی عملکرد سه منعقد کننده مختلف جهت تصفیه شیرابه کارخانه کمپوست اصفهان

http://hsr.mui.ac.ir/index.php/jhsr/article/view/727

مقدمه: در بسیاری از کشورهای جهان شیرابه زباله بدون هیچ تصفیه‌ای در محیط رها می‌شود و به دلیل ترکیب متفاوت شیرابه زباله در مکان‌های مختلف، تاکنون روش یکنواختی برای آن ارایه نشده است. عدم کنترل و بی‌توجهی نسبت به تصفیه و دفع صحیح شیرابه موجب آلودگی محیط می‌گردد. هدف از این مطالعه، مقایسه کارایی حذف COD (Chemical oxygen demond) و TSS (Total suspended solids) شیرابه حاصل از کارخانه کمپوست اصفهان با استفاده از منعقد کننده‌های سولفات آهن، کلرور آهن و پلی‌فریک سولفات و تعیین مناسب‌ترین منعقد کننده و کمک منعقد کننده بود. روش‌ها: در این مطالعه که از نوع مداخله‌ای بود، حذف COD و TSS از شیرابه کارخانه کمپوست اصفهان با استفاده از منعقد کننده‌ها و کمک منعقد کننده‌های مختلف مورد بررسی قرار گرفت. در این تحقیق غلظت‌ها و میزان pH‌های مختلف برای هر یک از منعقد کننده‌ها آزمایش گردید و مناسب‌ترین منعقد کننده در این شرایط تعیین شد. تحقیق با استفاده از روش آزمایش جار بررسی گردید و میزان TSS با استفاده از روش B-5210 و COD با استفاده از روش D-2540 استاندارد اندازه‌گیری شد. یافته‌ها: حداکثر راندمان حذف COD با استفاده از دوز g/l 2 سولفات فرو در 10 = pH، 43 درصد و TSS در دوز g/l 5/2، 38 درصد بود. در دوز g/l 5/1 کلرورفریک در 10 = pH، 31 درصد حذف COD و در دوز g/l 2، 47 درصد حذف TSS و در دوز g/l 2 پلی‌فریک سولفات در 11 = pH، 54 درصد حذف COD و در دوز g/l 5/2 پلی‌فریک سولفات، 51 درصد حذف TSS مشاهده شد. مناسب‌ترین کمک منعقد کننده برای کلرور‌فریک و پلی‌فریک سولفات 913SNF-AN بود که به ترتیب 41 و 58 درصد حذف COD داشت و در مورد سولفات فرو، کمک منعقد کننده پریستول با حذف 51 درصد COD بیش‌ترین راندمان حذف را دارا بود. نتیجه‌گیری: در حذف COD و TSS مؤثرترین منعقد کننده پلی‌فریک سولفات بوده است که در pH بازی و دوز g/l 2 نتیجه بهتری را نشان می‌دهد. بر اساس نتایج به دست آمده می‌توان روش انعقاد و کواگولاسیون را روشی مناسب و ارزان قیمت جهت پیش‌تصفیه شیرابه حاصل از کارخانه کمپوست اصفهان و آماده سازی آن برای تصفیه بهتر و مناسب‌تر با روش‌های دیگر معرفی نمود.

Title: مقایسه کفش‌های طبی با شاخص‌های ارگونومی و بررسی تأثیر این کفش‌ها بر عملکرد و کاهش دردهای عضلانی

http://hsr.mui.ac.ir/index.php/jhsr/article/view/307

مقدمه: کفش طبی راه‌هایی برای درمان ساختمان و عملکردهای مربوط به مشکلات پا را ارایه می‌کند. اشخاص با پای سالم نیازی به کفش طبی ندارند. کفش‌ها دارای طرح‌ها و ارتفاعات متفاوتی هستند و ممکن است با مواد مختلفی بسته به ماهیت تصحیحات لازم ایجاد می‌شود . سفارش یک کفش طبی مانند دارو مخصوص و ویژه هر شخص است. با این حال امروزه شرکت‌های زیادی محصولات خود را به عنوان کفش طبی به فروش می‌رسانند و افراد بدون آگاهی از نوع بیماری و بدشکلی و بدون نظر پزشک از این کفش‌ها به عنوان ابزار درمانی استفاده می‌کنند. هدف از مطالعه حاضر، بررسی تطابق کفش‌های طبی با شاخص‌های ارگونومی و تأثیر آن‌ها بر عملکرد فرد در قسمت کف پا، کمر و زانو بود. مواد و روش‌ها: در این بررسی ابتدا 4 نوع از کفش‌های موجود در بازار که به عنوان کفش طبی عرضه می‌شود انتخاب و با شاخص‌های ارگونومی کیفی مقایسه گردید و در مرحله دوم تأثیر این کفش‌ها بر تسکین دردهای عضلانی و افزایش عملکرد فرد در قسمت کف پا، کمر و زانو بررسی شد. یافته‌ها: هیچ کدام از کفش‌های انتخابی به طور کامل با استانداردهای ارگونومی تطابق نداشتند. همچنین تأثیر این کفش‌ها بر تسکین درد و افزایش عملکرد فقط در قسمت کف پا معنی‌دار بود. نتیجه‌گیری: در این مطالعه مشخص شد که کفش‌های عرضه شده به عنوان طبی حتی به طور کامل با شاخص‌های عمومی مربوط به کفش هم تطابق ندارند و استفاده از آن‌ها به عنوان ابزار درمانی مناسب نیست. در نتیجه آگاه کردن افراد جامعه در این رابطه می‌تواند مفید باشد. همچنین لزوم تدوین استاندارد و نظارت کافی بر تولید کفش به ویژه کفش‌های طبی، امری ضروری به نظر می‌رسد. انجام مطالعات مشابه برای جمع‌آوری اطلاعات بیشتر پیرامون این کفش‌ها پیشنهاد می‌شود.
